# First report of blood parasites in fishes from Kashmir and their effect on the haematological profile

**Published:** 2013-09-04

**Authors:** N. Shahi, A.R. Yousuf, M.I. Rather, F. Ahmad, T. Yaseen

**Affiliations:** 1 *Centre of Research for Development, University of Kashmir, Srinagar, India*; 2 *Department of Environmental Science, University of Kashmir, Srinagar, India*; 3 *Department of Zoology, University of Kashmir, Srinagar, India*

**Keywords:** *Trypanosoma mukasai*, *Babesiosoma*, Fish parasites, Hematology, Kashmir waters

## Abstract

*Cyprinus carpio communis* Linnaeus, *Carassius carassius* Linnaeus, *Schizothorax curvifrons* Heckel and *Triplophysa marmorata* species of fishes were captured from Anchar Lake and river Jhelum of Kashmir Himalaya for hematological and parasitological analysis. During the investigation haemoflagellates from the genus *Babesiosoma* and *Trypanosoma* were recorded in the blood smears. Trypanosomes were present in all the species except *C. carpio*, whereas *Babesiosoma* were only found in *T. marmorata*. Haematological analysis revealed a significant (*p*<0.01) reduction in red blood cell count in the fishes infected with *Babesiosoma* and *Trypanosoma*. A significant decrease (*p*<0.05) was recorded in haemoglobin value and packed cell volume in the infected fishes in comparison to the non-infected fishes.

## Introduction

*Babesiosoma* are parasites that inhabit both circulating erythrocytes and erythrocytes from reticulo-endothelial tissues and complete their life cycle in two hosts i.e., a vertebrate host (fish) and an invertebrate host (Leeche). Members of the genus *Babesiosoma* are intra-erythrocytic parasites whose hosts include frogs, toads, newts and fish (both marine and freshwater). In the vertebrate (intermediate) hosts, *Babesiosomes* form four merozoites during merogony and gamonts, usually from cruciform or rosette-shaped meronts (Barta, 1991; Lom and Dykova, 1992). Barta (1991) reported six dactylosomatids of the genus *Babesiosoma* from fishes, although three of these species were subsequently assigned to the genus *Haemohormidium* Henry (Siddall *et al.*, 1994).

Trypanosomes are haemoflagellates having a single free flagellum at the anterior end of their body. The first trypanosome was discovered from the blood of *Salmo trutta* by Valentin (1841). Since then, the parasite has been reported in fishes from different parts of the globe. For example, *T*. *mukasai*, *T*. *froesi*, *T*. *satakei* and *T*. *britskii* from Brazil (Lopes *et al.*, 1991), *T*. *occidentalis* from Washington (Becker, 1967) and *T*. *acanthobramae* and *T*. *neinevana* were recorded from Iraq (Warsi and Fattohy, 1976).

From India, Qadri (1962) reported *T*. *batrachi* from *Clarias batrachus*; *T*. *gachuii* from *Ophiocephalus gachua* (Misra *et al.*, 1973); *T*. *elongatus* from *Channa punctatus* (Raychaudhuri and Misra, 1973); *T*. *armeti* from *Mastacembelus armatus* (Mandal, 1978); *T*. *trichogasteri* (Gupta and Jairajpuri, 1981), *T*. *colisi* (Gupta, 1986), *T*. *trichogasteri* var. fasciatae (Gupta *et* *al*., 1998) and *T*. *piscidium* (Gupta *et al.*, 2003) from *Colisa fasciata*; *T*. *rohilkhandae* (Gupta and Saraswat, 1991) and *T*. *sauli* (Gupta *et al.*, 2006) from *Channa punctatus*.

Most species of trypanosomes infecting fishes cause pathogenic diseases of considerable medical and economic importance. Symptoms of piscine trypanosomiasis range from mild anemia associated with low levels of parasitaemia to severe pathological changes due to a heavy parasite burden (Islam and Woo, 1991). Leukocytosis, hypoglycemia and hypocholesterolemia are the frequent outcomes of trypanosomiasis (Gupta and Jairajpuri, 1983).

Although a great number of parasitological investigations have been conducted on fishes in Kashmir, most of the data pertain to ecto and endo-parasites mainly associated with the digestive system (Kaw, 1950, 1951; Fotedar, 1958; Fotedar and Dhar 1973, 1974, 1977). The piscine haemoparasites and haemtological profile have not been investigated so far. Thus, the present study is aimed to identify and report the blood parasites of the genus *Trypanosoma* and *Babesiosoma* parasitizing freshwater fishes in Jhelum River and Anchar Lake, Kashmir. In addition, the haematological profiles of both infected and uninfected fishes were analysed.

## Materials and Methods

The study area was Anchar Lake and River Jhelum in Kashmir. Live fish belonging to four taxa namely, *Cyprinus carpio* Linnaeus, *Carassius carassius* Linnaeus, *Schizothorax curvifrons* Heckel and *Triplophysa marmorata* (Heckel, 1838) were captured from River Jhelum (34°04’17’’N/74°49’08’’E) and Anchar Lake (34°08’48’’N/74°47’22’’E) monthly between December, 2008 to June, 2009. On the field, the identification of the fish and the collection of samples were carried out. Blood was collected from 210 live fishes from the caudal peduncle and heart as described by Lucky (1977). Part of the blood sample was used to make smears on grease-free glass slides for staining. For determining haematology, blood samples were collected into glass vials containing EDTA as an anticoagulant at an approximate concentration of 5mg/ml of blood (Blaxhall and Daisley, 1973).

Thin blood smears were made from the blood samples collected. The smears were air dried and fixed in absolute methanol. Slides were stained with Phosphate buffered Geimsa and examined under a microscope using a 100x oil immersion objective. Images were taken with the help of a Leica DM LS2 digital camera. Measurements were made as described by Lom and Dykova (1992).

The haemoglobin (Hb) content of the blood samples was estimated by cyanomethemoglobin method (Brown, 1980). The number of red and white blood cells was determined using a Neubauer Chamber. The packed cell volume (PCV) was determined using Wintrobe’s tube method according to Ramnik (1994), while the mean cell volume (MCV) and the, mean cell haemoglobin concentration (MCHC) were obtained according to the method given by Dacie and Lewis (2001).

For physico-chemical analysis, water samples were collected from five sites each in the Anchar Lake and the River Jhelum on a monthly basis during December, 2008 to June 2009. The water samples were collected in polythene bottles just below the surface of the water during morning hours. Water temperature was recorded with the help of Celsius thermometer having 0.1 ºC precision. For estimating the Dissolved Oxygen content, the water was fixed in 250ml Biological Oxygen Demand bottles on the spot. Analysis of other parameters was done in the laboratory within 24 hrs in accordance with the guidelines of APHA (1998) and CSIR (1974). Data were analyzed using ANOVA and Student’s t-test.

## Results

**Fig. 1:**
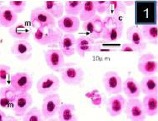
Babesiosoma sp. in a Giemsa-stained blood film from different fishes in River Jhelum and Anchar Lake at 1000X; **1**) undivided meronts (m) and cruciform meront (c); **2)** elongated gamonts (g); **3)** undivided meronts (m) and elongated gamonts (g).

**Fig. 2:**
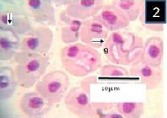
Babesiosoma sp. in a Giemsa-stained blood film from different fishes in River Jhelum and Anchar Lake at 1000X; **1**) undivided meronts (m) and cruciform meront (c); **2)** elongated gamonts (g); **3)** undivided meronts (m) and elongated gamonts (g).

**Fig. 3:**
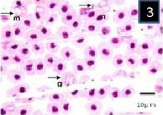
Babesiosoma sp. in a Giemsa-stained blood film from different fishes in River Jhelum and Anchar Lake at 1000X; **1**) undivided meronts (m) and cruciform meront (c); **2)** elongated gamonts (g); **3)** undivided meronts (m) and elongated gamonts (g).

**Fig. 4:**
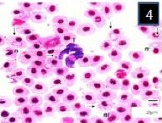
Three different morphological forms of *Trypanosoma mukasai* and simultaneous infection of *babesioma* and *T. mukasai* in a Giemsa-stained blood film from *Triplophysa marmorata* in Anchar Lake; **4)** *T. mukasai* (t) undivided and meronts (m) and merozoites (mr) (m); **5)** T. mukasai (t) and undivided meronts (m); **6)** Broad gamonts.

**Fig. 5:**
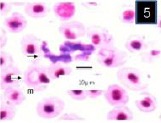
Three different morphological forms of *Trypanosoma mukasai* and simultaneous infection of *babesioma* and *T. mukasai* in a Giemsa-stained blood film from *Triplophysa marmorata* in Anchar Lake; **4)** *T. mukasai* (t) undivided and meronts (m) and merozoites (mr) (m); **5)** T. mukasai (t) and undivided meronts (m); **6)** Broad gamonts.

**Fig. 6:**
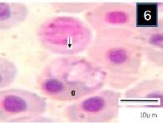
Three different morphological forms of *Trypanosoma mukasai* and simultaneous infection of *babesioma* and *T. mukasai* in a Giemsa-stained blood film from *Triplophysa marmorata* in Anchar Lake; **4)** *T. mukasai* (t) undivided and meronts (m) and merozoites (mr) (m); **5)** T. mukasai (t) and undivided meronts (m); **6)** Broad gamonts.

In Giemsa-stained blood films, various stages of babesiosome parasite were found intraerythrocytic (Figs 1-6). These stages were identified as meronts, merozoites and gamonts. Displacement of the nucleus in the infected erythrocytes was detected in all of these stages (Figs. 1-6). Undivided meronts (Figs. 1 and 3-6) usually occurred singly within erythrocytes, but two meronts were noted occasionally. They were oval or elongated, and measured 4.5±0.44 µm long by 2.6±0.39 µm wide (parasitized cell size of 9.8±2.01 × 7.8±0.72 µm). The central area of each meront was non-staining, with the entire periphery of the meront stained deep purple, as with chromatin. *Babesiosoma* was found to divide into four meronts in a cross-like pattern. Cruciform meronts (Fig. 1) measured 5.3±0.58 µm long on each of the long axes of the cross and 4.3±0.58 µm wide (parasitized cell size of 9±0 × 6±0 µm). Chromatin was most noticeable at the four extremities of the cross in the cruciform meronts (divided meronts).

Merozoites, presumably arising from cruciform meronts, were usually found in fours within red blood cells (Fig. 4). These were elongated with rounded ends, with staining properties which were generally similar to those of meronts, and measured 3±0.82 µm long by 2.7±0.5 µm wide (parasitized cell size of 9±0.05 × 9±0.05 µm). While the Gamonts were sometimes rather broad (Fig. 9), but were mostly elongate with one end slightly more swollen than the other which was usually pointed (Figs. 2 and 3). Gamonts measured 5.3±0.64 µm long by 3.1±0.47 µm wide (parasitized cell size of 9.2±1.59 × 9.6±1.43 µm). Its margins were clearly defined and stained deep blue but the nucleus was not distinguishable (Figs. 2, 3 and 9). *Babesiosoma* were observed to infect *T. marmorata* with a prevalence of 16.6%.

**Fig. 7:**
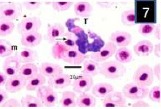
Trypanosoma mukasai in a Giemsa-stained blood film from different fishes in River Jhelum and Anchar Lake at 1000X; **7)** *Triplophysa marmorata*; **8)** *Carassius carassius*; **9)** *Schizothorax curvifrons*.

**Fig. 8:**
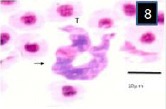
Trypanosoma mukasai in a Giemsa-stained blood film from different fishes in River Jhelum and Anchar Lake at 1000X; **7)** *Triplophysa marmorata*; **8)** *Carassius carassius*; **9)** *Schizothorax curvifrons*.

**Fig. 9:**
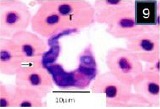
Trypanosoma mukasai in a Giemsa-stained blood film from different fishes in River Jhelum and Anchar Lake at 1000X; **7)** *Triplophysa marmorata*; **8)** *Carassius carassius*; **9)** *Schizothorax curvifrons*.

In blood films, the body of the trypanosome stained deep blue, though its free flagellum, arising from the pointed anterior end of the body, was poorly stained. The kinetoplast was prominent, lying close to the blunt posterior end of the body (Figs. 7-9). The rounded nucleus stained pink with Giemsa and was closer to the anterior end of the trypanosome than to its posterior extremity. Morphometry and the number of fishes analyzed during are shown in Table 1.

**Table 1:** Morphometry and the number of fishes analyzed during the present study.

Type of Fish	No. of sampled fishes	Length (centimeters)	Weight (grams)
*Cyprinus carpio*	55	21.73±2.15	190.83±53.99
*Carassius carassius*	55	14.39±2.17	58.99±31.85
*Schizothorax curvifrons*	60	23.05±3.16	128.05±58.11
*Triplophysa marmorata*	40	6.53±1.51	91.5±10.5

**Table 2:** Measurements of Trypanosoma mukasai (values expressed as Mean ±S.D) in μm from the fishes of Anchar lake and River Jhelum.

Measurement	Length (µm)
Body length (BL)	36.63±4.35
Body width (BW)	4.64±0.88
Nuclear length (NL)	5.12±1.01
Nuclear width (NW)	3.86±0.5
Middle of nucleus to anterior extremity (AN)	14.42±2.62
Posterior extremity to middle of nucleus (PN)	21.71±3.1
Nuclear index (NI=PN/AN)	1.54±0.34
Kinetoplast to middle of nucleus (KN)	20.53±3.5
Kinetoplast Index (KI = PN/KN)	1.12±0.03
Total length (TL)	47.13± 3.1
Flagellar index (FI)	3.4

**Table 3:** Overall Prevalence of *Trypanosoma mukasai* and *Babesiosoma sp*. in different fishes collected from Anchar Lake and River Jhelum.

Parasite	Fish	Water body	Prevalence (%)
*T. mukasai*	*Triplophysa marmorata*	Anchar Lake	41.6
	*Carassius carassius*	Anchar Lake	2.32
	*Schizothorax curvifrons*	River Jhelum	7.6
	*Cyprinus carpio scapularis*	Anchar Lake	0
	*Cyprinus carpio communis*	Anchar Lake	0
	*Cyprinus carpio scapularis*	River Jhelum	0
	*Cyprinus carpio communis*	River Jhelum	0
*Babesiosoma* sp.	*Triplophysa marmorata*	Anchar Lake	16.6%

**Table 4:** Haematological parameters (Mean ± S.D) of the infected fishes

Parameters	Infected fish	
	*Carassius carrasius*	*Schizothorax curvifrons*
RBC (10^6^/mm^3^)	0.53±0.19	0.98±0.67
WBC (10^3^/mm^3^)	88.98±77.43	245.65±180.76
Hb (gm)	6.4±3.21	7.32±5.21
PCV	24.52±6.72	24.85±7.46
MCV (µm^3^)	510.74±151.12	328.2±197.04
MCH (µg)	124.37±60.37	69.53±43.25
MCHC (%)	23.507±9.20	23.5±11.16

**Table 5:** Haematological parameters (Mean ± 0 S.D) of the non-infected fish.

Parameters	Non-infected fish			
	*Carassius carassius*	*Schizothorax curvifrons*	*Cyprinus carpio communis*	*Cyprinus carpio specularis*
RBC (10^6^/mm^3^)	1.1 ±0.06	2.2 ±0.18	0.98 ±0.67	0.72 ±0.18
WBC (10^3^/mm^3^)	35.0 ±5.0	2.3 ±0.51	245.65 ±180.76	19 ±1.4
Hb (gm)	7.9 ±0.1	10.4 ±0.74	7.32 ±5.21	7.32 ±4.09
PCV	29 ±0.82	32.8 ±2.27	24.85 ±7.46	19.67 ±4.50
MCV (µm^3^)	270.8 ±17.93	146.6 ±2.28	328.2 ±197.04	273.67 ±42.06
MCH (µg)	73.7 ±3.70	43.6 ±4.01	69.53 ±43.25	112.33 ±78.45
MCHC (%)	27.2 ±0.87	35.4 ±3.55	23.5 ±11.16	43.67 ±36.47

RBC-Red blood cell, WBC-White blood cell, Hb- haemoglobin, PCV-packed cell volume, MCV- Mean cell volume, MCH- Mean cell Haemoglobin, MCHC- Mean cell Haemoglobin concentration.

**Table 6:** Mean values of Physico- Chemical characters of water from Anchar Lake and River Jhelum.

Parameters	Anchar Lake	River Jhelum
Water Temp. (°C)	13.4±5.54	12 ±5
Depth (m)	0.6±0.47	1.09±0.6*
pH	7.4±0.45	7.01±0.2*
Transparency (m)	0.5±0.32	0.39±0.2*
Conductivity (µS)	222.5 ±63.5	118.3±17.6*
Dissolved Oxygen (mg/l)	3.8±0.39	4.54±0.7*
Free CO_2_ (mg/l)	3.1±1.24	2.86±1.3
Alkalinity (mg/l)	207.8±71.79	133.1±37.0*
Chloride (mg/l)	33.1±7.62	27.8±7.4**
Total Hardness (mg/l)	184.6±51.12	122.8±45.2*
Ammonia (µg/l)	360.38±207.87	70.6±5. 13*
Nitrite-N (µg/l)	29.56±16.23	32.3±6.7
Nitrate-N (µg/l)	411.38±158.43	211.5±134.9*
Total Phosphorus (µg/l)	433.3±162.1907	236.1±57.7*
Ortho phosphorus (µg/l)	177.0±84.1	42.5±15.4*

* Significantly different from Anchar Lake at *p*< 0.001

** Significantly different from Anchar Lake at *p*<0.05

## Discussion

The higher prevalence of infection of *T*. *mukasai* and *Babesiosoma* sp. in *Triplophysa* *marmorata* when compared to other fishes in the same water bodies (Anchar Lake) seems to be attributable to the habitat preferences of these fish. *Triplophysa marmorata* may spend a lot of its time near or within vegetation, and this may make them highly exposed to infection and reinfection by leech bites. Leeches, once engorged with the blood of the host, detach and rest on a protected substrate (preferably under a stone or in plant debris) in the water until their next meal (Paperna, 1996). This makes *T. marmorata* more prone to trypanosome infection than other fishes from the same habitat. Piscine *Dactylosoma* divides into 4, in cross-like pattern, or into 8 in an octagonal formation. Dactylosomatids with only quadruple division were regarded as a separate genus *Babesiosoma* (Jakowska and Nigrelli, 1956), while those dividing into 8 were named *Haemohormidium* (Khan, 1980) but it appears that the same species had alternating generations forming either 4 or 8 progeny (Paperna, 1996). However, during the present study it seemed that Dactylosoma does not have alternating generations as otherwise stated by Paperna (1996) and it was however; found that *Babesiosoma* had only quadruple division.

Baker (1960) found two morphological forms *of Trypanosoma mukasai* - small (22-44μm long) and large (45-65μm) - in the blood of fish. Baker (1960) commented that nuclear position, rather than the flagellar length may be important in distinguishing the African freshwater fish trypanosomes. The nucleus lies forward of the mid-line consistently in the trypanosomes recorded in this study (see NI values in Table 2), which support their identity as *T. mukasai*.

Letch and Ball (1979) suggested that the anemia in fish is more the result of repeated feedings of leeches (the vector), than a direct effect of the protozoan infection. Infected *S*. *curvifrons* showed relatively higher number of lymphocytes than non-infected ones. Khan (1985) experimentally infested *Gadus morhua* with *Trypanosoma murmanensis* (Nikitin, 1927), and observed low hematocrit and haemoglobin concentration in the infected fish. Anemia was the most common aspect. Positively correlated with parasitism, the anemic fish are lethargic. In the light of present study, the significant decrease in the RBCs, haemoglobin and haematocrit of fishes from Anchar Lake in comparison to those from River Jhelum may be in part due to higher concentration of nitrite 32.3±6.7 µg/l in Anchar Lake than 29.56±16.23 µg/l in River Jhelum which leads to fluctuation in blood parameters.

It could be inferred that physiological response to nitrite is an increase in methemoglobin content. The hemoglobin becomes oxidized and is unable to bind and carry oxygen molecules (Brown and McLeay, 1975). Nitrite enters the fish from the gills and enters the circulatory system (Perrone and Meade, 1977). Fish with elevated levels of methemoglobin may suffer from anoxia (Huey *et al.*, 1980; Tomasso, 1981). When the methemoglobin content of the blood exceeds 70 to 80 % of the total hemoglobin, fish becomes torpid, unresponsive and disoriented (Klinger, 1957).

The reduction in RBC count, haemoglobin value and packed cell volume in the infected fishes occurred as a result of the parasitic infestation that often leads to anemia (Martins *et al.*, 2004). Furthermore, the parasites simply act as a stressor and during primary stages of stress the packed cell volume is altered due to the release of catecholamine, which can mobilize RBCs from spleen (Wells and Weber, 1990) or induce RBCs to swell as a result of fluid entry into the intracellular compartment (Chiocchia and Motais, 1989). Similar results were recorded by Hassen (2002) and Ismail (2003) in *Clarias garipienus* that are naturally infected with *T*. *mukasai*.

Decreased total leucocyte count in response to pollutant exposure has been observed by Singh and Srivastava (1992), Singh and Srivastava, (1994) and Pandey and Pandey (2001). A similar trend in fish of Anchar Lake and River Jhelum was observed. Pulsford (1984), in case of *Scyliorhinus canicola* infected by trypanosomes found a reduction of Hematocrit and an increase in the number of leucocytes. Similar results were found in *S. curvifrons*. This can be attributed to decline in *Schizothorax* Heckel species in water bodies.
